# Experimental Test of Heat Treatment Effect on Physical Properties of Red Oak (*Quercus falcate* Michx.) and Southern Pine (*Pinus taeda* L.)

**DOI:** 10.3390/ma7117314

**Published:** 2014-11-05

**Authors:** Derya Sevim Korkut, Salim Hiziroglu

**Affiliations:** 1 Department of Forest Industry Engineering, Duzce University, 81620 Duzce, Turkey; E-Mail: deryasevimkorkut@duzce.edu.tr; 2 Department of Natural Resource Ecology and Management, Oklahoma State University, Stillwater, OK 74078, USA

**Keywords:** heat treatment, Southern red oak, Southern pine, surface roughness, swelling

## Abstract

The objective of this work was to evaluate the effect of heat treatment and compression on the swelling and surface roughness of Southern red oak (*Quercus falcate* Michx.) and Southern pine (*Pinus taeda* L.). Specimens were exposed to temperature levels of 110 °C or 200 °C for 8 h before they were compressed using 2.5 MPa pressure for 5 min. Swelling values of the control and heat-treated samples in three grain orientations were evaluated by soaking them in water for 48 h. A stylus method was employed to determine the surface characteristics of the samples. Three main roughness parameters, namely mean arithmetic deviation of profile (*R*_a_), mean peak-to-valley height (*R*_z_), and maximum roughness (*R*_max_) were used to evaluate the effect of heat treatment on surface characteristics of the samples. Oak and pine specimens had 39.8% and 28.7% lower tangential swelling values, respectively, than those of control samples as a result of exposure to a temperature of 200 °C. Heat treatment did not make any significant difference on surface quality. Micrographs taken from cross sections of the specimens revealed that there was some cell distortion and modification due to heat treatment as well as compression. Combination of heat treatment and compression can be considered an alternative method to improve certain physical properties of these two species.

## 1. Introduction

Red oak and Southern pine are two species widely used for many applications, including general construction. Southern red oak is the common name for *Quercus falcata* Michx., which is a native species in the Southern United States [1]. Red oak is also one of the main species used to manufacture furniture and cabinets in North America. Southern pine (*Pinus taeda*) is classified as Southern yellow pine, which is a native softwood species distributed within the Coastal Plain, from the west to Eastern Texas and Southeastern Oklahoma [2]. *P. taeda* wood has a density of 0.47 g/cm^3^, maximum tangential shrinkage value of 7.66% and excellent mechanical properties with an average modulus of rupture of 734 kg/cm^2^ and modulus of elasticity of 100,200 kg/cm^2^ [2,3].

Although wood is an excellent renewable building material, it has some disadvantages, such as its degradation under outdoor conditions and dimensional instability as a function of the fluctuation of relative humidity in the surrounding environment. Therefore, it is important to treat wood using various methods so that not only it can be used effectively during its service life but also its dimensional stability can be enhanced. Thermal treatment is one of the methods used to improve the dimensional characteristics of wood and wood products. Thermal treatment of wood has been investigated since the middle of the last century for the purpose of avoiding the toxic effects of chemical treatments to improve the properties of wood [4].

Thermal treatment consists of applying heat to wood or wood products at temperatures between 100 and 250 °C, depending on the degree of modification that is desired [5]. During heat treatment, a large number of chemical changes occur, including the esterification of hydroxyl groups and reduction of hemicellulose and the number of accessible OH groups within wood. Consequently, heat treatment can serve to improve the natural quality and properties of the wood, including dimensional stability and resistance to biological deterioration. However, heat treatment generally results in an apparent reduction of mechanical properties of the members due to material losses in the cell wall, hemicellulose degradation and the modification of long chain molecules. Previous experiments showed that heat treatment decreases the equilibrium moisture content and thickness swelling and slowed down the water absorption as well as wettability of various wood species [6].

Although most of the physical properties of the two species considered in this work have been investigated in past studies, there is little or no information on the wood characteristics as a function of different heat treatment schedules and compression in the literature. The main objective of this study was to evaluate some basic properties, including oven-dry density, surface roughness, and swelling, of Southern red oak and Southern pine species as a function of different heat treatment schedules compression. Therefore, this work was performed to yield an initial data on the dimensional stability and surface quality of samples from two species as a result of combination of heat treatment and compression so that the species can be used more effectively and efficiently for the manufacture of different value-added products.

## 2. Experimental

### 2.1. Materials and Methods

**Figure 1:**
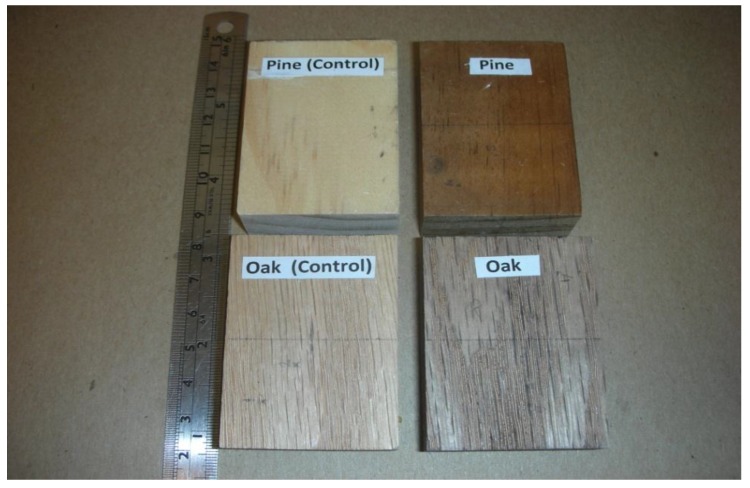
Heat-treated and control samples of pine and oak.

Two species, namely Southern red oak (*Quercus falcate* Michx.) and Southern pine (*Pinus taeda* L.) samples supplied by a local sawmill in Oklahoma were used in this work. A total of 72 defect-free samples with dimensions of 40 mm by 55 mm by 19 mm from each of the two species were conditioned in a room with a temperature of 20 °C and a relative humidity of 65% until they reached an equilibrium moisture content of 12%. Dimensions of the samples were also measured and weighed to an accuracy of 0.01 mm and 0.01 g, respectively, before and after heat exposure to calculate their dimensional and moisture content changes. Specimens exposed to temperature levels of 110 °C or 200 °C for 8 h in addition to control samples used for the experiment. Heat treatment of the samples was carried out in a laboratory-type oven controlled at an accuracy of ±1 °C under atmospheric pressure. In the next step; treated specimens were compressed on a Carver press for 5 min using a pressure of 2.5 MPa. Figure 1 illustrates typical control samples and those exposed to a temperature of 200 °C for 8 h. Heat-treated samples along with control samples were soaked in distilled water for 48 h to determine their swelling values. All measured samples were also kept in the oven at 103 ± 2 °C for 24 h to determine their moisture content values.

There are various methods to evaluate the surface quality of wood and wood products, but the stylus method was successfully used to evaluate surface roughness both with solid wood and wood composite samples in past studies [7,8]. Eight measurements with a tracing span of 15.2 mm were taken from both sides of each sample across the grain orientation by employing a Hommel T-500 profilometer (Hommel America Inc., New Britain, CT, USA) before and after heat treatment and compression of the samples. The stylus unit used in this study consisted of the main unit and the pick-up model TkE. The pick-up had a skid-type diamond stylus with a 5-µm tip radius and 90° tip angle [9]. Three well-accepted roughness parameters that can be calculated from digital information obtained from the surface of each sample, *i.e.*, average roughness (*R*_a_), maximum roughness (*R*_max_) and mean-peak-to-height (*R*_z_), were used to evaluate the surface quality of the samples [10].

The effect of heat treatment on the anatomical structure of the sample was also investigated using a scanning electron microscope (SEM, New York, NY, USA) JEOL Model Quanta. Samples of 5 mm × 5 mm × 5 mm were coated with a thin layer of gold in the vacuum chamber of a sputter coater for 2 min. Micrographs were taken from cross sections of the control and heat treated samples exposed to various treatment schedules. Analysis of variance (ANOVA) was performed using Statistical Analysis System (SAS) software (Detroit, MI, USA) to determine the significant differences of all parameters used in this study.

## 3. Results and Discussion

**Figure 2:**
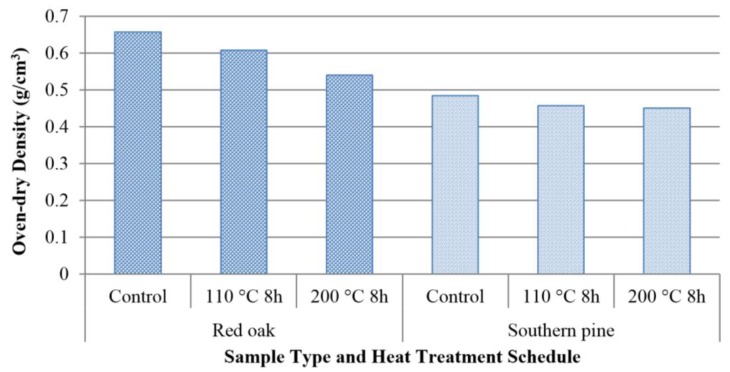
Oven-dry density values of the samples.

Table 1 displays the test results of oak samples. Control specimens had an average oven-dry density of 0.657 g/cm^3^, and this value was reduced 7.45% and 17.80% as a result of heat exposure at temperature levels of 110 °C and 200 °C, respectively. The corresponding weight losses for pine specimens were 5.80% and 9.27%, respectively. Figure 2 illustrates the oven-dry density values of both species.

Degradation of hemicellulose into volatile substances and evaporation of extractives are considered the main parameters responsible for the density reduction of wood exposed to heat [11]. In this work, in addition to density reduction, the specimens also had weight loss, which increased with increasing magnitude of temperature, as can be seen in Table 1 and Table 2. The weight loss of spruce exposed to a temperature of 225 °C for 6 h was 12.5% [12]. The overall findings in our study are relatively similar to those determined in past study [12]. It can be concluded that the weight loss in samples heat-treated at higher temperatures is mainly due to the degradation of hemicelluloses and cellulose.

**Table 1:** Test results of heat-treated red oak specimens.

Heat treatment	Exposure time	Oven-dry density (g/cm^3^)	Weight loss *WL* (%)	Roughness parameters			Swelling		
				*R*_a_ (µm)	*R*_z_ (µm)	*R*_max_ (µm)	Radial (%)	Tangential (%)	Longitudinal (%)
Control	0 h	0.657 a (0.070) * *12*	–	9.97 a (5.97) * *72*	37.42 a (14.44) * *72*	101.41 a (39.72) * *72*	2.63 a (0.69) * *12*	4.69 a (1.28) * *12*	0.27 a (0.12) * *12*
110 °C	8 h	0.608 a (0.080) * *12*	5.80	12.82 b (6.23) * *72*	36.97 a (17.11) * *72*	119.92 b (29.04) * *72*	3.81 b (0.51) * *12*	5.67 b (1.48) * *12*	0.20 b (0.05) * *12*
200 °C	8 h	0.540 b (0.078) * *12*	9.27	10.96 a (4.70) * *72*	43.76 a (13.85) * *72*	124.85 b (28.60) * *72*	2.03 c (0.26) * *12*	2.82 c (0.36) * *12*	0.17 b (0.12) * *12*

Notes: * Values in parentheses are standard deviation. Italic values are number of samples used in each test; Groups with the same letters—a,b,c in each column indicate that there is no statistical difference (*p* < 0.05) between the samples according to Duncan’s multiple range test.

**Table 2:** Test results of heat-treated southern pine specimens.

Heat treatment	Exposure time	Oven-dry density (g/cm^3^)	Weight loss *WL* (%)	Roughness parameters			Swelling		
				*R*_a_ (µm)	*R*_z_ (µm)	*R*_max_ (µm)	Radial (%)	Tangential (%)	Longitudinal (%)
Control	0 h	0.484 a (0.059) * *12*	–	3.00 a (0.56) * *72*	23.34 a (4.10) * *72*	33.45 a (10.90) * *72*	2.92 a (0.75) * *12*	4.84 a (0.56) * *12*	0.37 a (0.09) * *12*
110 °C	8 h	0.457 a (0.057) * *12*	7.44	2.89 a (0.73) * *72*	22.93 a (5.10) * *72*	28.90 b (8.74) * *72*	3.62 b (0.47) * *12*	5.63 b (0.49) * *12*	0.29 b (0.09) * *12*
200 °C	8 h	0.451 a (0.056) * *12*	9.23	2.93 a (0.57) * *72*	23.18 a (5.55) * *72*	34.68 a (15.79) * *72*	3.22 a (0.53) * *12*	3.45 c (0.73) * *12*	0.15 c (0.10) * *12*

Notes: * Values in parentheses are standard deviation. Italic values are number of samples used in each test; Groups with the same letters—a,b,c in each column indicate that there is no statistical difference (*p* < 0.05) between the samples according to Duncan’s multiple range test.

Control samples of Southern red oak had average values of 2.63% radial and 4.69% tangential swelling as a result of the water soaking test. It appears that exposure to a temperature of 200 °C for 8 h enhanced their dimensional stability, giving lower swelling characteristics. The radial and tangential swelling of the samples were reduced 22.81% and 39.87% as compared to control samples. Similar trends were also observed for pine samples, as displayed in Table 2.

However, pine samples had 10.27% and 28.72 radial and tangential swelling values, respectively, which are lower than those of oak specimens. This could be related to the less porous anatomical structure and lower density of pine as compared to oak species. Springback and release of the compression stresses within the samples could also be responsible for the high swelling values for both oak and pine samples.

It appears that s positive effect of heat treatment on the dimensional stability of the samples is still clear, even if they were compressed prior the water soaking. It is a well-known fact that lignin, hemicellulose and cellulose change their chemical structures, which prevents water reabsorption to some degree due to their tendency to attach between as well as within the polymers of wood [13].

**Figure 3:**
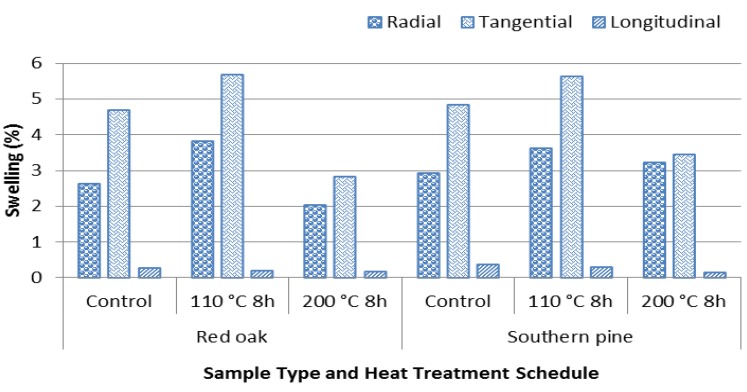
Swelling values of control and heat-treated samples.

Both oak and pine specimens considered in this work had slightly increased swelling values in all three grain orientations when they were exposed to a temperature of 110 °C for 8 h. This could be due to initial stress relief of the compressed samples. However, it appears that stress relief of the specimen was compensated with increased temperature to 200 °C, so that their dimensional stability was enhanced as can be seen Figure 3.

In a past study, it was found that Sitka spruce samples exposed to a temperature of 320 °C had 60% reduced swelling values [14]. Živković *et al.* [15] investigated the dimensional stability of heat-treated wood samples and determined that beech and ash samples treated at a temperature of 190 °C had 35% and 27% improvement in their dimensional stability, respectively.

During heat treatment, the content of hemicellulose, which is relatively easy to hydrolyze at elevated temperature, significantly decreases with increasing exposure duration and temperature. The crystallinity content of wood can also be increased due to crystallization in quasicrystalline regions in wood cellulose and even in hemicelluloses. In addition, the esterification of hydroxyl groups and cross-linking reactions occurs during heat treatment of wood. Based on the combined effects of these factors, the OH groups available for moisture adsorption are significantly reduced by heat treatment, which in turn decreases the hygroscopicity and equilibrium moisture content of wood. In all of the above factors, the decrease in the hemicellulose content of wood is usually believed to be the main factor in the decrease of the hygroscopicity of wood [16].

**Figure 4:**
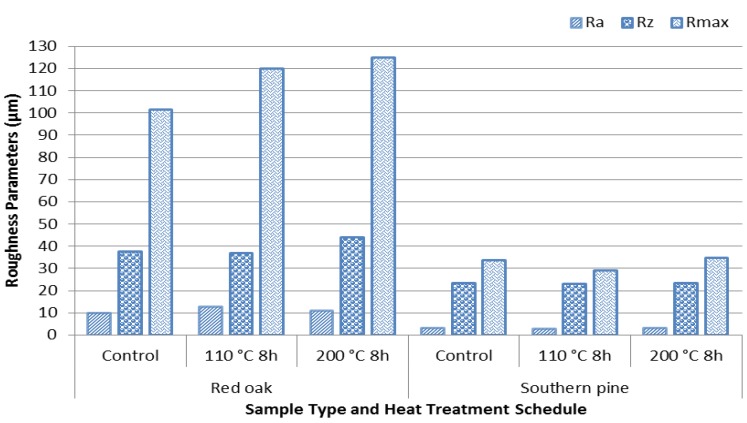
Roughness values of control and heat-treated samples.

The pine control sample had a relatively lower surface roughness than oak samples, with an average *R*_a_ value of 3.00 µm *versus* 9.97 µm. The substantial porous structure of oak is responsible for its rough surface. Heat exposure and compression of both types of samples did not show any significant changes on their surface quality as shown in Figure 4.

A previous work revealed that a temperature of 200 °C for 8 h did not cause any substantial changes on surface roughness of the specimens [17]. It seems that unless the temperature is increased beyond 200 °C and the samples exposed to heat for more than 8 h, would not have any major changes on their surface quality.

**Figure 5:**
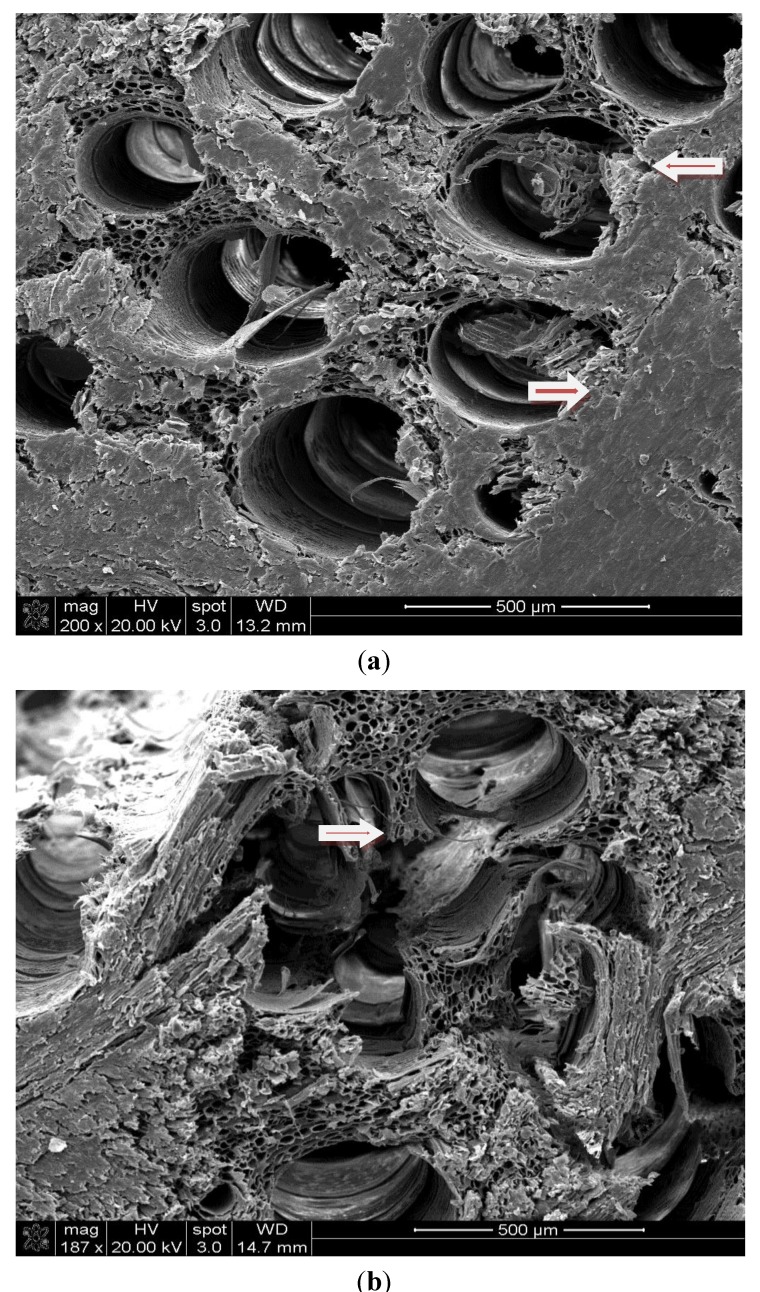
(**a**) Undamaged cell wall of control Southern red oak sample; (**b**) Damaged cell wall of Southern red oak sample exposed to a temperature of 200 °C for 8 h.

It is a known fact that heat treatment reduces the overall mechanical properties of wood [18]. The strength and stiffness of wood decrease with increasing temperature and exposure time [19]. If wood is exposed to increased temperatures for an extended time, its strength properties are permanently decreased [20]. It was not the objective of this work to determine the effect of heat treatment on mechanical properties of the samples. However, micrographs were taken from the cross section of the specimen to observe if the combination of heat treatment and compression would cause any changes in their microstructure. Figure 5 is typical micrographs illustrating the cell wall of the cross sections of control Southern red oak specimens and those exposed to a temperature of 200 °C. It is quite clear that high temperature in combination with compression resulted in certain damage to the cells in the form of breaking the cell wall as illustrated in Figure 5b. It can be concluded that although compression increased the overall density of wood, when it is combined with heat treatment, the samples became brittle. Heat-treated wood species without any compression also had some cracks and distortions in the cell wall, as determined in a previous work [17].

## 4. Conclusions

Within the scope of this investigation surface roughness quality, dimensional stability in three grain orientations and oven-dry density of compressed Southern pine and Southern red oak samples were evaluated as a function of heat treatment.

It appears that overall swelling of the samples improved as a result of heat treatment. However, the surface roughness of both types of samples did not show any significant change due to heat exposure.

With their enhanced dimensional stability as a result of heat treatment, Southern red oak and Southern pine could have potential to be used in different value-added applications including flooring and window frames more effectively.

This is an ongoing study and next phase of the investigation will include applying different heat treatment schedule to the samples from two species under various compression levels. In addition to dimensional movement of the samples their water absorption as function of two combined processes will also be evaluated. In further studies effect of above parameters on the chemical composition and combustion properties of two species would also be interesting to gain a better understanding of their behavior.
